# A Year's Work of the Ministry of Health

**Published:** 1921-09-03

**Authors:** 


### THE PUBLIC WELL-BEING.

### A Year's Work of the Ministry of Health.

The process of sorting-out and redistributing the extremely complicated functions which come under the scope of the Health Minister can only gradually be completed. Every year progress is recorded in the task of defining what does and what does not come within the purview of the Minister, and the work thus accomplished, though less interesting to the public than definite reduction of disease, has a most important bearing on the health of the community. A very large proportion of the waste complained of by the ratepayer is due, we suspect, to nothing more sensational than the usurpation of powers by public bodies unfitted to exercise them efficiently. In the recently issued Report of the Ministry of Health reference is made to the transfer to the Ministry from the Home Office of certain work under the Anatomy Acts; of various powers and duties of far-reaching importance in relation to mental disease; and of the enforcement of certain provisions of the Factory and Workshop Act, 1901, including notably the prohibition of the employment of women for a statutory period after child-birth. The Ministry has disembarrassed itself of certain other duties, such as work in relation to gas undertakings of local authorities, etc., and has accomplished a fine piece of devolution in the establishment of the Welsh Board of Health.

It is largely by its success in the treatment of tuberculosis, venereal, and other infectious diseases that the Department is likely to be judged, but the administrative changes which took place last year owing to the passing of the National Health Insurance Act, 1920, render comparisons difficult with previous years. The County and County Borough Councils have now the onus of providing institutional treatment for insured persons suffering from tuberculosis direct, as well as for non-insured persons under the tuberculosis schemes of various areas, and all but one of these Councils (the exception is Great Yarmouth) have submitted schemes for the approval of the Minister of Health which are now being carried out. In all 418 institutions containing 17,352 beds are in working order, and the number of approved dispensaries has risen to 411, an increase of thirteen in the year. The advance of after-care and training colonies has suffered from what the report alludes to as "the general financial stringency," but nine institutions in various parts of the country already afford training facilities for some 1,100 men. In the development of this branch of treatment lies the key to prevention no less than cure. Local authorities have entered with vigour into the campaign against venereal disease, and that some impression is being made would seem to be evidenced by the rise of attendances at treatment centres from 488,000 in 1918 to 1,489,000 in 1920. The large proportion of patients, more than half the total number, who ceased to attend before completing the course of treatment was, however, very disappointing.

The Ministry of Health has to its credit a very useful and humane piece of work in the registration of the blind and the extension of the old-age pension to such as at the age of fifty were prevented by defects of vision from earning their own living. This measure has removed a vast amount of wretchedness and anxiety from a most unfortunate class in the community at a comparatively small cost. The total number of blind persons registered up to March 31, 1921, is 35,020, and of these 7,826 have been awarded pensions, 93.4 per cent. at the maximum rate of 10s. a week. An attempt was made during the past year to collect and publish comparative figures of the weekly cost per head of maintaining inmates in Poor-Law institutions, and some extraordinary variations were revealed. In one district the cost per head varied from £1 5s. 5d. per week to 10s. 3d. We await with much interest returns for the year ending March 31, 1920. They are stated to show an increase of some 50 per cent. over the figures obtainable for the preceding year, due mainly to the increase in cost of living and of officers' salaries and rations. The Ministry has been compelled to draw the attention of the Guardians to the existing legal obstacles which prohibit the treatment of non-Poor-Law patients in Poor-Law institutions, but the way is left open for co-ordinating any scheme with other local agencies for the provision of medical treatment, the division of functions between local institutions, and the provision of facilities for medical education in the Poor-Law establishments. We notice with regret that the process of getting the children out of the workhouses is still retarded. On January 1 last year there were 2,674 healthy children in workhouses, of whom 799 were in the general wards. On January 1, 1921, the number had only been reduced to 2,157, of whom 676 were in general wards. The time has come when this defective organisation should no longer be tolerated.

